# Hexavalent chromium elimination from wastewater by integrated micro-electrolysis composites synthesized from red mud and rice straw via a facile one-pot method

**DOI:** 10.1038/s41598-022-18598-7

**Published:** 2022-08-20

**Authors:** Huabin Wang, Ting Cui, Dingxiang Chen, Qiong Luo, Jiwei Xu, Rong Sun, Wenhua Zi, Rui Xu, Ying Liu, Yong Zhang

**Affiliations:** 1 School of Energy and Environment Science, Yunnan Normal University, Kunming, 650500 People’s Republic of China; 2 Yunnan Key Laboratory of Rural Energy Engineering, Kunming, 650500 People’s Republic of China

**Keywords:** Environmental sciences, Environmental chemistry, Pollution remediation

## Abstract

The widely spread chromium (Cr) contamination is rising environmental concerns, while the reutilization of agro-industrial by-products are also urgently demanded due to their potential risks. In this study, we prepared the integrated micro-electrolysis composites (IMC) through a facile one-pot method with red mud and rice straw. The effects of components relatively mass ratios as well as pyrolysis temperature were analyzed. The XRD, XPS, SEM, FTIR, and various techniques proved the IMC was successfully synthesized, which was also used to analyze the reaction mechanisms. In this study, the dosage of IMC, pH, adsorption time, and temperature of adsorption processes were explored, in the adsorption experiment of Cr(VI), dosage of IMC was 2 g/L (pH 6, 25 °C, and 200 rpm) for isothermal, while the concentration and contact time were also varied. According to the batch experiments, IMC exhibited acceptable removal capacity (190.6 mg/g) on Cr(VI) and the efficiency reached 97.74%. The removal mechanisms of adsorbed Cr(VI) were mainly elaborated as chemical reduction, complexation, co-precipitation, and physical adherence. All these results shed light on the facile preparation and agro-industrial by-products recycled as engineering materials for the heavy metals decontamination in wastewater.

## Introduction

The widely-spread contamination caused by heavy metals raised increasing concerns in past few decades^1–3^. These heavy metal pollutants, such as chromium (Cr) species, exhibited low mobility and poor biodegradability, which are toxic to aquatic lives as well as human beings^4,5^. The conventional existed chemical states of Cr species are Cr(VI) and Cr(III), while the former one exhibited more toxicity to human’s kidney, brain, and other apparatus^6,7^. Many approaches were applied to solve the problem of Cr(VI) contamination, including physical filtration, flocculation, bio-remediation, and adsorption^8^. Among these approaches, the adsorption demonstrated advantages of high efficiency, neglect second pollution, cost-effectiveness, and facile operation^9^. The core issue for adsorption process was the design and application of adsorbents, and there is urgently demand on the effective adsorbents for the Cr(VI) decontamination^10,11^.

Recently, integrated micro-electrolysis composites (IMC) have been designed and employed for the heavy metal pollution control. These IMC materials could conduct the micro-electrolysis reactions between inherent components and environmental contaminants, forming the integrated oxidative reactions which attracted research attention. Further, with the advantages of high chemical reactivity, stability, and availability, IMC prepared by various feedstocks could apply in many practical agro-industrial situations^12,13^. Many raw materials are employed for IMC synthesis, including graphic nanotubes, modified clays, carbonaceous nanofibers, and biochar functionalized with nano-particles^14^. The cost-effective biomass derived IMC could adhere pollutants, and various methods were conducted to the fabrication of bio-adsorbents, including gas activation, bacterial attachment, and chemical modifications^15^. Among them, loading with iron species including Fe^0^, Fe_3_O_4_, Fe_2_O_3_, is considered as a potential solution for IMC performance improvement, due to the existence of active iron species, especially for Fe^0^ could provide excellent chemical reactivity for IMC^16^.

The active Fe^0^ species modified onto the carbonaceous substrate to form IMC not only could enhance the performance of composites^17^, and the existence of biochar also could protect Fe^0^ oxidation by air and prevent the aggregation of iron species^18^. Our group prepared IMC functional materials from red mud and rice straw for the removal of heavy contaminants, and offered evidence for chemically reduction by Fe^0^ in IMC^19^. Further proved the feasibility of this functional materials for the heavy metal elimination^20,21^. However, the method for synthesis biochar fabricated with Fe^0^ to form IMC was generally complex and time-consuming, normally biochar was first pyrolysis under oxygen-limited conditions, and then, loading with iron slats (Fe(II) or Fe(III)), finally, the reductive chemicals, such as NaBH_4_, were applied to reduce iron species to Fe^0^. Therefore, more facile and cost-effective method should be designed to improve the practicality of this IMC materials.

Nowadays, hydrothermal treatment is widely used as a proficient method for biomass fabrication, due to the facile operation and mild condition. The homogeneous dispersion of components could react under the high temperature and self-generated pressure, and followed pyrolysis process could transfer hydrochar into biochar^22^. Moreover, our group found that during thermal conversion, the generated reductive gas (H_2_ and CO) from black liquor could reduce Fe(II) or Fe(III) to Fe^0^, which is more facile compared to conventional preparation methods^23,24^.

Furthermore, the iron salts to prepare IMC was iron source, while the red mud, generated during aluminum industry, contained huge amount of iron species (> 30 wt%) could act as iron source for IMC preparation^25,26^. This hypothesis could provide a solution for IMC synthesis, and also provide a feasible and promising approach for recycling and reutilization of this industrial byproduct.

Hence, in this work, the IMC materials were synthesized from agro-industrial byproducts via a facile hydrothermal and subsequently pyrolysis method, and then employed for the removal of Cr(VI) from wastewater. The objectives of this study were: (1) Using industrial by-product red mud and rice straw, preparation of zero-valent iron-rich IMC by one-pot method; (2) characterizing the physiochemical and morphological properties of obtained IMC; (3) analyzing the effects of IMC on hexavalent chromium removal under different preparation process parameters and obtaining the optimal IMC with the best removal efficiency. (4) Investigating the surface interactions between IMC and Cr(VI) via various models; (5) elucidating the removal mechanisms of this IMC material through various techniques".

## Results and discussion

### Physiochemical characterization

**Figure 1:**
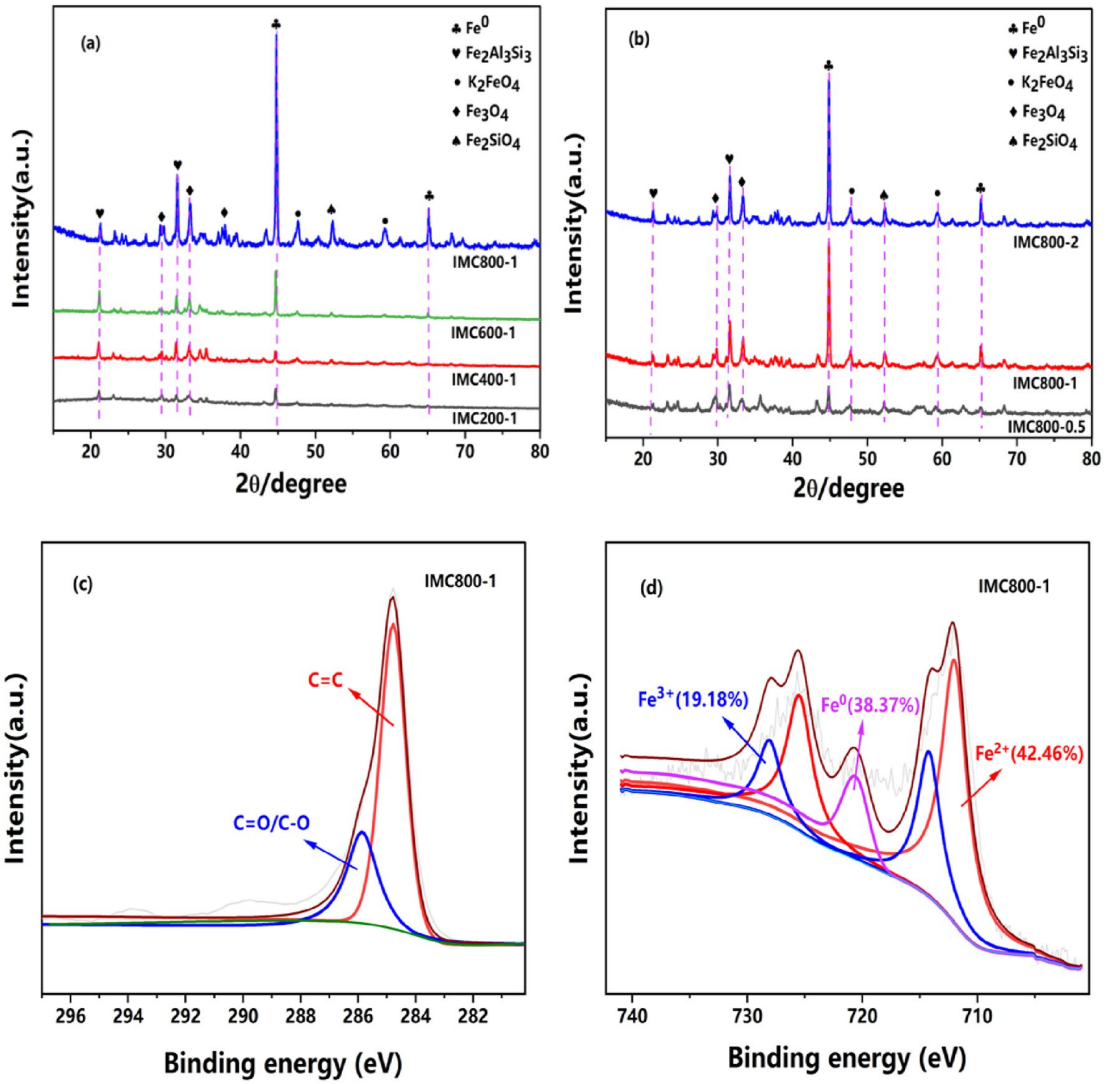
XRD patterns (**a**), (**b**) and the high-resolution C 1 s, Fe 2p spectra of IMC (**c**), (**d**).

The crystalline structure of IMC and other materials was investigated by XRD, as shown in Fig. 1a. After hydrothermal and co-pyrolysis, the distinctive peaks were observed in IMC800-1, with the 2θ of 44.7° and 65.0°, demonstrating the variations of the iron chemical states and existence of Fe^0^ (PDF#03-065-4899)^27^. However, these peaks were neglectable in the samples of IMC200-1 and IMC400-1, indicating the pyrolysis temperature acted as essential roles for the formation of Fe^0^ species, and lower temperature (< 600 °C) could not form IMC contained Fe^0^. The formation of Fe^0^ from biomass and red mud could be explained by the generated hydrogen and CO from biomass could reduce Fe^2+^ and Fe^3+^ to Fe^0^, and the observed Fe_3_O_4_ with the 2θ of 29.8° and 33.4° (PDF#00-034-0417) also proved the transforming processes^28,29^. Meanwhile, the existence of Fe_2_Al_3_Si_3_, Fe_2_SiO_4_, and K_2_FeO_4_, could be attributed to the inherent impurities in the red mud and straw biomass. The influence of different ratios was also analyzed as shown in Fig. 1b. The obvious peaks of Fe^0^ were detected in the spectra of IMC800-2 and IMC800-1, and weak crystalline peaks in IMC800-0.5, this phenomenon could be explained by the more biomass in IMC precursors could improve the transformation of iron species, leading to the more complete conversion of Fe. Hence, after this facile one-pot treatment with carbothermal reduction process, the biomass converted into biochar, and iron species turned to Fe^0^, forming the IMC materials, which could contribute the reduction and adsorption of target pollutants^30^.

The chemical states of surface elements in IMC were further investigated by XPS, as depicted in Fig. 1c,d. The revolution of chemical states of C and Fe could elucidate the reactions during IMC formation. The peaks of C 1 s (Fig. 1c) with the binding energy of 284.78 eV and 284.78 eV related to the structure of C=C and C=O/C–O^31,32^. These results could be contributed to the generation of oxygen-contained functional groups during pyrolysis processes. In the case of Fe 2p peaks, the binding energy with 720.78 eV indicating the successfully preparation of Fe^0^ in IMC after pyrolysis (Fig. 1d). The existence of these Fe^0^ species (with the molar ratio of 38.37%) was in a good agreement with XRD spectra, which were chemically reactive for the pollutant’s elimination. Meanwhile, the presence of Fe^2+^ and Fe^3+^ was also proved with the binding energy of 711.68 and 725.48 eV for Fe 2p_3/2_, as well as 713.08 eV and 717.28 eV for Fe 2p_1/2_, respectively^33^. These could be explained by the incomplete transformation of iron oxides into chemically reactive states. Hence, according to aforementioned results, we could find that the IMC with reactive species through this facile one-pot hydrothermal and subsequent co-pyrolysis process, these could be expressed as following Eq. (1):

1 $${\text{Fe}}_{2} {\text{O}}_{3} + {\text{C}}_{{\text{x}}} {\text{H}}_{{\text{y}}} {\text{O}}_{{\text{z}}} \left( {{\text{in }}\,\,{\text{intermediate }}\,\,{\text{products }}} \right) \to {\text{C}}_{{{\text{x}} - 3}} {\text{H}}_{{{\text{y}} - 2}} {\text{O}}_{{\text{z}}} + 2{\text{Fe}}_{0} + {\text{CO}} + {\text{H}}_{2}$$

**Figure 2:**
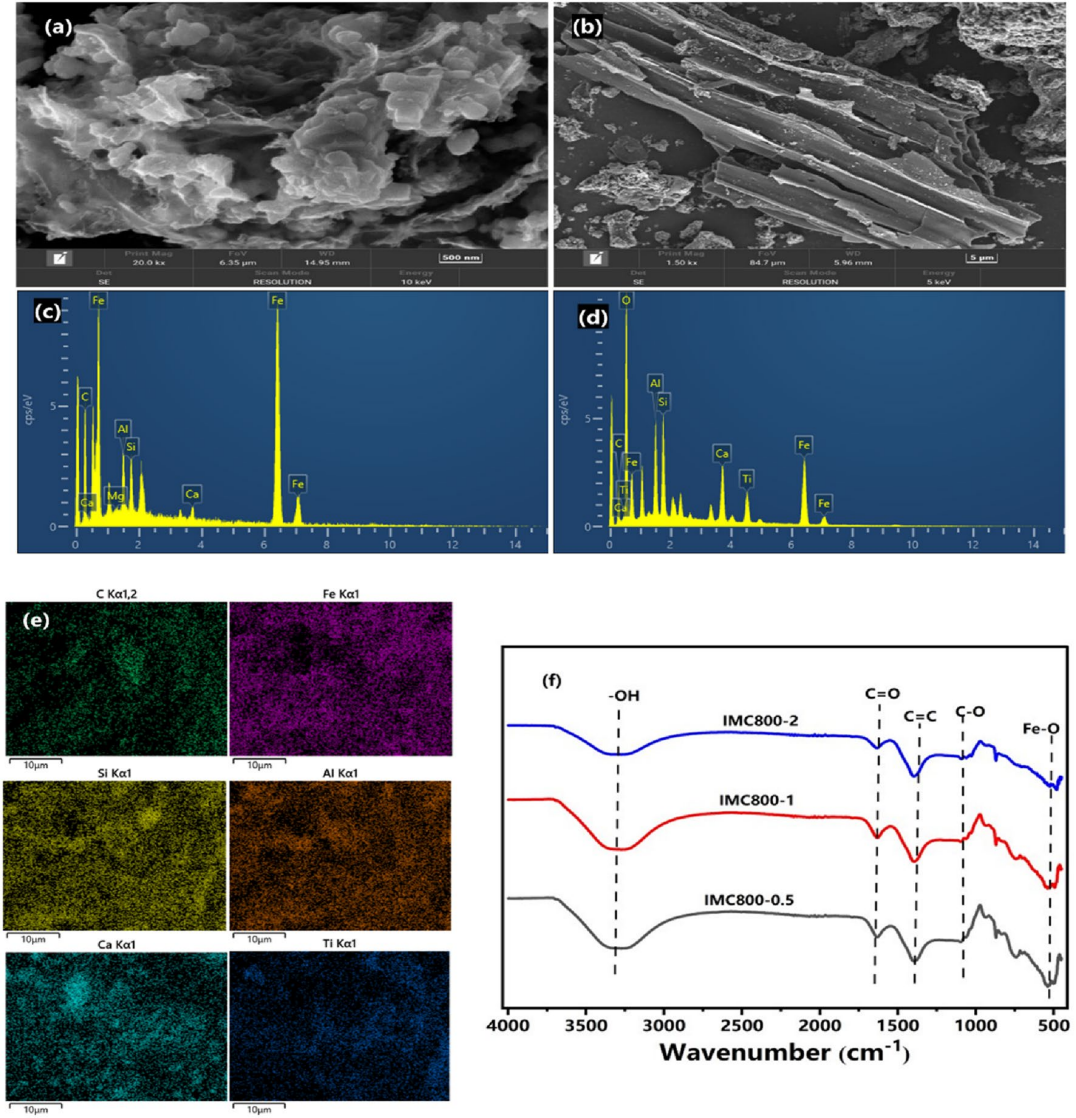
SEM–EDS analysis of IMC (**a**–**e**), Fourier transform infrared spectra (FT-IR) at different ratios of IMC800 (**f**).

The surface morphologies and elemental distribution of different IMC were observed by SEM–EDX (Fig. 2). As shown in results, the irregular pores and unsatisfied porous configurations in IMC800-0.5 were confirmed. After loading with more iron species from red mud, in the SEM results of IMC800-1, the presence Fe^0^ was proved with spheres structure, moreover, the wrinkled surface was damaged, and more pore structure occurred^34^. This phenomenon might be attributed to the catalytic properties of iron species during biomass pyrolysis^35^. And these generated pores provided more reactive sites for pollutants. Interestingly, according to the EDS results are exhibited in Fig. 2c–e, there were several minerals in IMC, such as Si, Ca, Al, and so on, these species inherited from red mud, and might could react with contaminants during reaction^28^. Hence, according to the SEM observation, the equally distributed Fe^0^ species further proved the successful synthesis of IMC materials.

The functional groups in IMC were also detected by FTIR, as shown in Fig. 2f. The wavenumber of 3432, 1632, and 1446 cm^−1^ was related to the vibration of –OH, C=O, and C–C, respectively^36,37^. These results demonstrated that the abundant functional groups on the IMC800 materials with various red mud loading rates. According to previous works, the peak with the wavelength of 553 cm^−1^ was attributed to the existence of Fe–O groups, while the peak at 1002 cm^−1^ ascribed to the stretching of C–O groups^38^. These results offered evidence that loading of iron-derived functional groups, more functional groups formed, despite some groups were occupied by the loading Fe^0^. Therefore, fabrication of Fe0-rich IMC, could also increase the amount of the functional groups, which might play essential roles during pollutants removal^39^.

All these physicochemical characterizations provided information that IMC was successfully prepared, with porous structure and abundant surface functional groups^40^. The optimal preparation conditions were the weight ratio between red mud to rice straw was 1:1 while co-pyrolysis with the temperature of 800 °C. And the Cr(VI) removal capacity was need to evaluate the performance of this IMC material.

### Batch adsorption experiments

**Figure 3:**
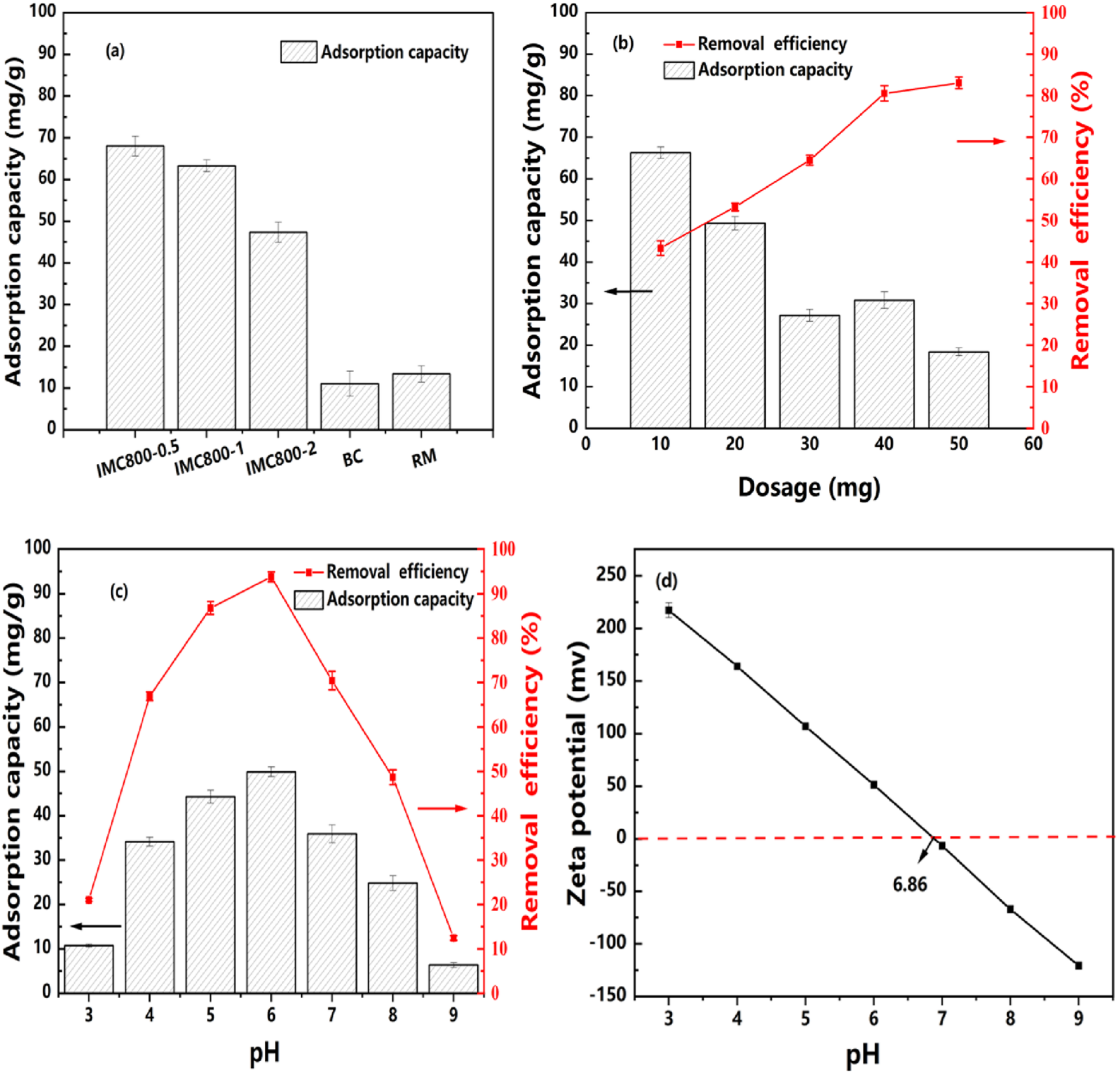
Removal capacity of various IMC materials (**a**), Influence of dosage on removing Cr(VI)of IMC (**b**), pH and Zeta potential determination (**c**), (**d**).

The Cr(VI) adsorption experiments were conducted to evaluate the removal capacity of various IMC materials. As shown in Fig. 3a, the removal capacity was increasing from 48.02 mg/g of IMC800-0.5 to the 68.03 mg/g of IMC800-2. These results could be explained the more loading of Fe^0^ in IMC. According to the former characterization, too much iron species from red mud was detrimental to the formation of species, due to lack of reductive biomass^41^. The removal capacity of BC800 and RM800 was relatively lower (nearly 10 mg/g), this further proved the functionality of IMC adsorbents.

The effects of adsorbents dosage were also investigated as shown in Fig. 3b. With the increasing of IMC800-1 dosage from 10 to 50 mg, the removal efficiency increased from 43.34 to 83.45%, while the capacity decreased from 66.75 to 19.51 mg/g. This could be attributed to the more adsorbents could compete with pollutants and the adsorption capacity for per unit adsorbents was inhibited. In order to balance removal efficiency and capacity, the dosage of 40 mg was applied for the further experiments.

The influence of solution initial pH during Cr(VI) removal by IMC was also investigated in Fig. 3c. As pH is considered as an essential factor influencing the surface interactions between adsorbent and contaminants, with the increasing of initial solution pH, the capacity of IMC firstly increased during pH 3 to 6, with the removal capacity from 11.05 to 51.66 mg/g. This phenomenon could be attributed to the chemically reduction from Cr(VI) to Cr(III) and followed adsorption on the IMC materials. However, with the continuous pH increment, the adsorption capacity decreased from 35.28 to 8.96 mg/g as the pH value changed from 7 to 9, as the efficiency changed from 70.14 to 14.98%. This could be explained by the existence of Cr(VI) in solution was mainly CrO_4_^2−^ species, which were electrostatic repulsion and competed for reactive sites with abundant OH^−^ species at high pH conditions^42^. In order to further elucidate the surface interactions under various pH, the zeta-potential experiments were also conducted and depicted in Fig. 3d. According to the tests, the zero-point -charge of pH (pH_ZPC_) was 6.86, indicating the negative charge when pH at alkaline conditions. These negatively charged surface further supported the analysis of experimental data. In other words higher pH leading to the negative charged IMC surface, which was detrimental to the Cr(VI) removal^42^. Therefore, in the following experiments, the pH 6 was chosen as initial solution pH values.

### Adsorption kinetics

**Figure 4:**
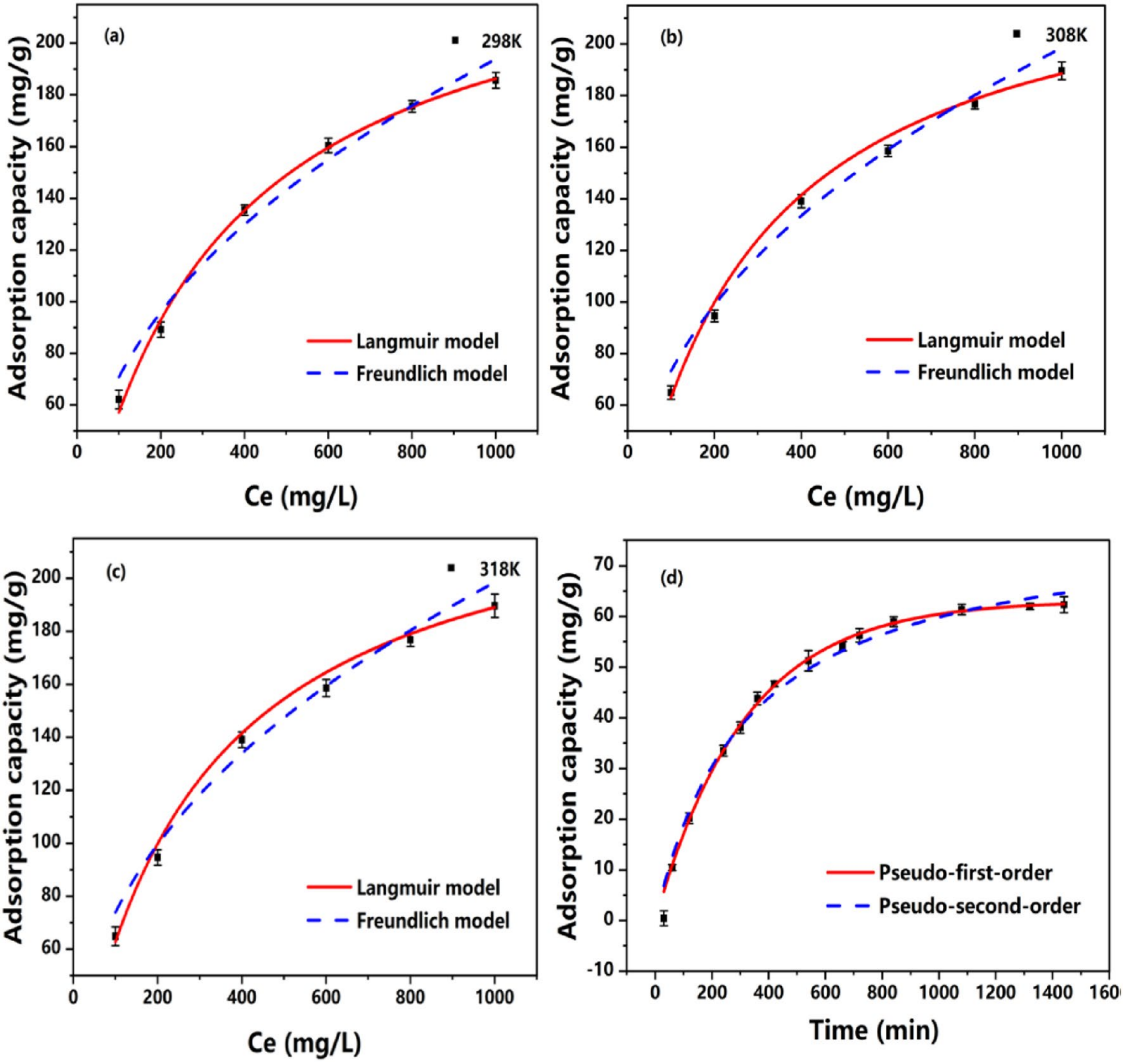
Influence of contact time on Cr(VI) sorption by IMC800-1 (**a**), the fit results of Langmuir and Freundlich models for IMC800-1 (**b**–**d**).

The interactive reactions between IMC and Cr(VI) also investigated by adsorption kinetics. As shown in Fig. 4a, the capacity of IMC was increased with the increment of time intervals, this could be attributed to the sufficient surface reactions between adsorbents and adsorbates. The reaction reached equilibrium after 720 min with the capacity of 56.3 mg/g. Several models were employed for the kinetics analysis of adsorption processes and the parameters were listed in Table 1. The fitting results indicated the pseudo-first-order fitted better compared with pseudo-second-order models, with the related coefficients (R^2^) were 0.999 and 0.991, respectively. But both models fit better, which was possibly the adsorption process is a coexistence of physical and chemical mechanisms^43^. These results demonstrated the existence of chemical bonding between adsorbents and contaminants, such as interactions with polar organic functional groups.

**Table 1:** Quasi-first-order and quasi-second-order kinetic model, isothermal and thermodynamics fitting parameters of IMC for Cr(VI) adsorption.

Adsorbent	Pseudo-first-order			Pseudo-second-order		
	q_e_ (mg/g)	k_1_ (min^−1^)	R^2^	q_e_ (mg/g)	k_2_ (g/(mg min))	R^2^
RMBC800-1	63.14	0.003	0.999	78.98	0.336	0.991

	Langmuir model			Freundlich model		
	q_m_ (mg/g)	K_L_ (L/mg)	R^2^	K_F_ (mg/g)	1/n	R^2^
298 K	248.83	0.002	0.996	9.44	0.437	0.971
308 K	242.32	0.003	0.994	9.94	0.433	0.986
318 K	243.57	0.003	0.995	10.24	0.429	0.984

	∆G° (kJ/mol)		∆H° (J/mol)		∆S°(J/(mol/k))	
298 K	− 5.67		6.52		10.24	
308 K	− 5.71					
318 K	− 6.12					

### Reaction isotherms

The surface interactions between adsorbates and IMC could be further analyzed by isotherm study, as shown in Fig. 4b–d. The different isotherm models were also employed to depict the surface interactions between adsorbents and target contaminants under various temperature conditions, and fitting results were listed in Table 1. The removal capacity of Cr(VI) increased gradually with the increasing of amount of pollutants, this could be attributed to the improved driving forces under high concentration. Freundlich model could interpret the adsorptive reactions with heterogeneous energetic distribution with multilayer active sites, while Langmuir model represent the sufficient homogeneous reactive sites with monolayer adsorption^44^. The related coefficients (R^2^) for Langmuir and Freundlich models were 0.996, 0.994, 0.995 and 0.971, 0.986, 0.984 at temperature of 298 K, 308 K, and 318 K, respectively. Hence, the Langmuir model cloud be more appropriate for mimicking the Cr(VI) immobilization, with the theoretical maximum capacity of 248.83, 242.32, and 243.57 mg/g for 298, 308, and 318 K. The fitting results hinted that Cr(VI) adsorption was in mainly monolayer with almost identical energetic sites. And the better results provided by Langmuir model represented that adsorption was monolayer process on heterogeneous reactive sites.

### Thermodynamic analysis

With the increasing of temperature, the sorption of Cr(VI) was improved, indicating the endothermic properties of this reactions. Cr(VI) was involved with the removal processes, represented as free energy, enthalpy, and entropy change, labelled as Δ*G*°, Δ*S*°, and Δ*H*°, respectively^45^. According to the results, the Cr(VI) removal was endothermic spontaneous, as listed in Table 1. These results also elucidated the multiple reactive mechanisms between ICM and pollutants, which would be explained in the mechanisms sections.

### Removal mechanisms

**Figure 5:**
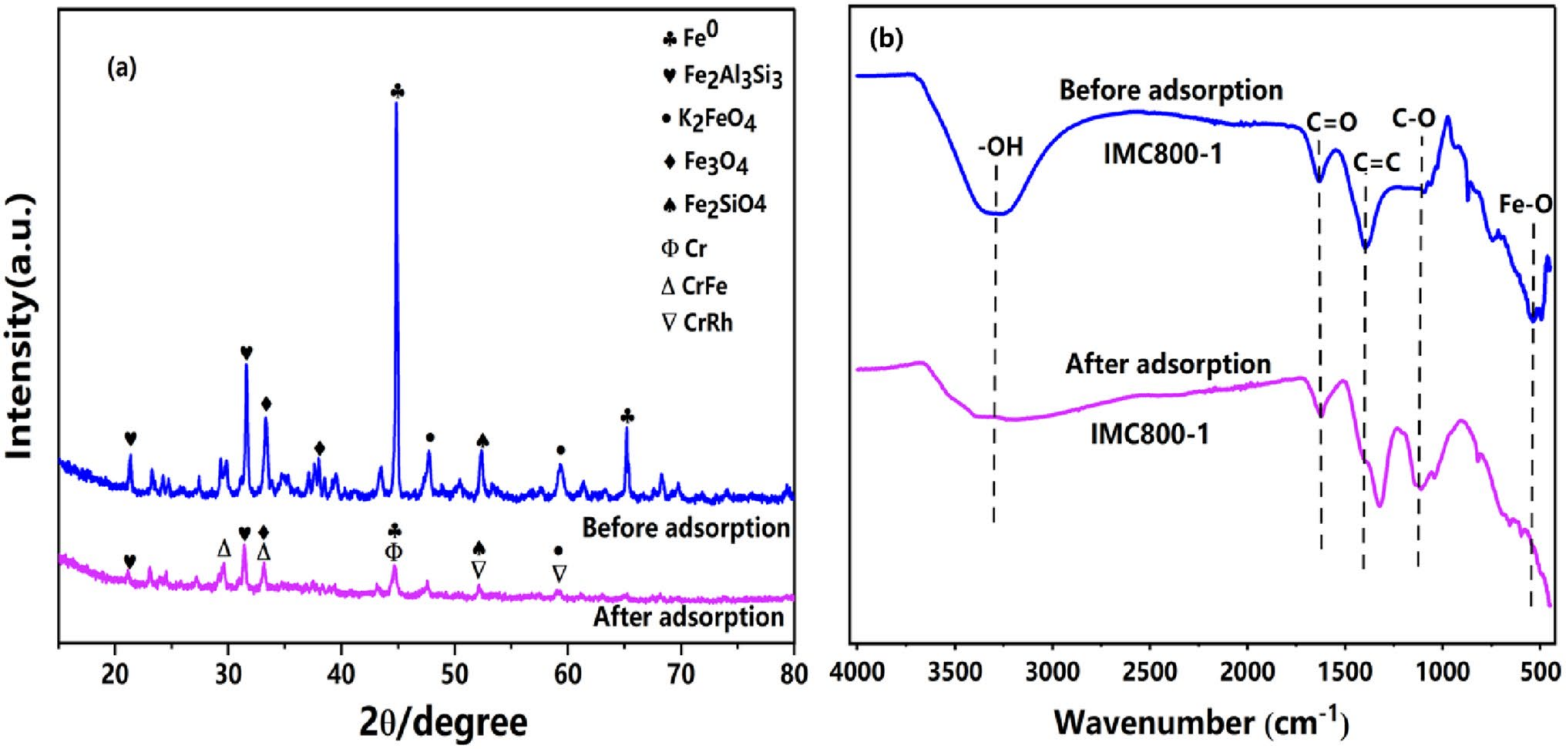
XRD patterns of IMC800-1 before and after adsorption (**a**), FTIR before and after adsorption (**b**).

Firstly, these mechanisms were analyzed by the XRD results, as shown in Fig. 5a. The complexation peaks with 2θ of 44.7° indicating the iron-containing substances in IMC participated in the adsorption process of Cr(VI), several impurities may also be participating in this procedure, and the corresponding diffraction peaks all have a decreasing trend. the peaks of Fe in IMC800-1 and was substantially weakened. In addition, compounds related to Cr also appeared indicating the formation of crystalline structure.

FTIR experiments was also proved the variations of surface functional groups, as shown in Fig. 5b. The weakened –OH vibration at 3432 cm^−1^ demonstrated that the functional groups were involved in the Cr(VI) removal, which could be reduction or formation of complexations^46^. The same phenomena were observed for the C=C and C–O stretching, indicating the different functional groups contributed to the Cr(VI) removal. with the following Eq. (2) by using hydroxyl groups as an example.

2 $$2 \equiv {\text{Fe}} - {\text{OH}} + {\text{CrO}}_{4}^{{2 - }} { \leftrightharpoons }2\left( { \equiv {\text{Fe}} - } \right)_{2} {\text{CrO}}_{4} + 2{\text{OH}}^{ - }$$

**Figure 6:**
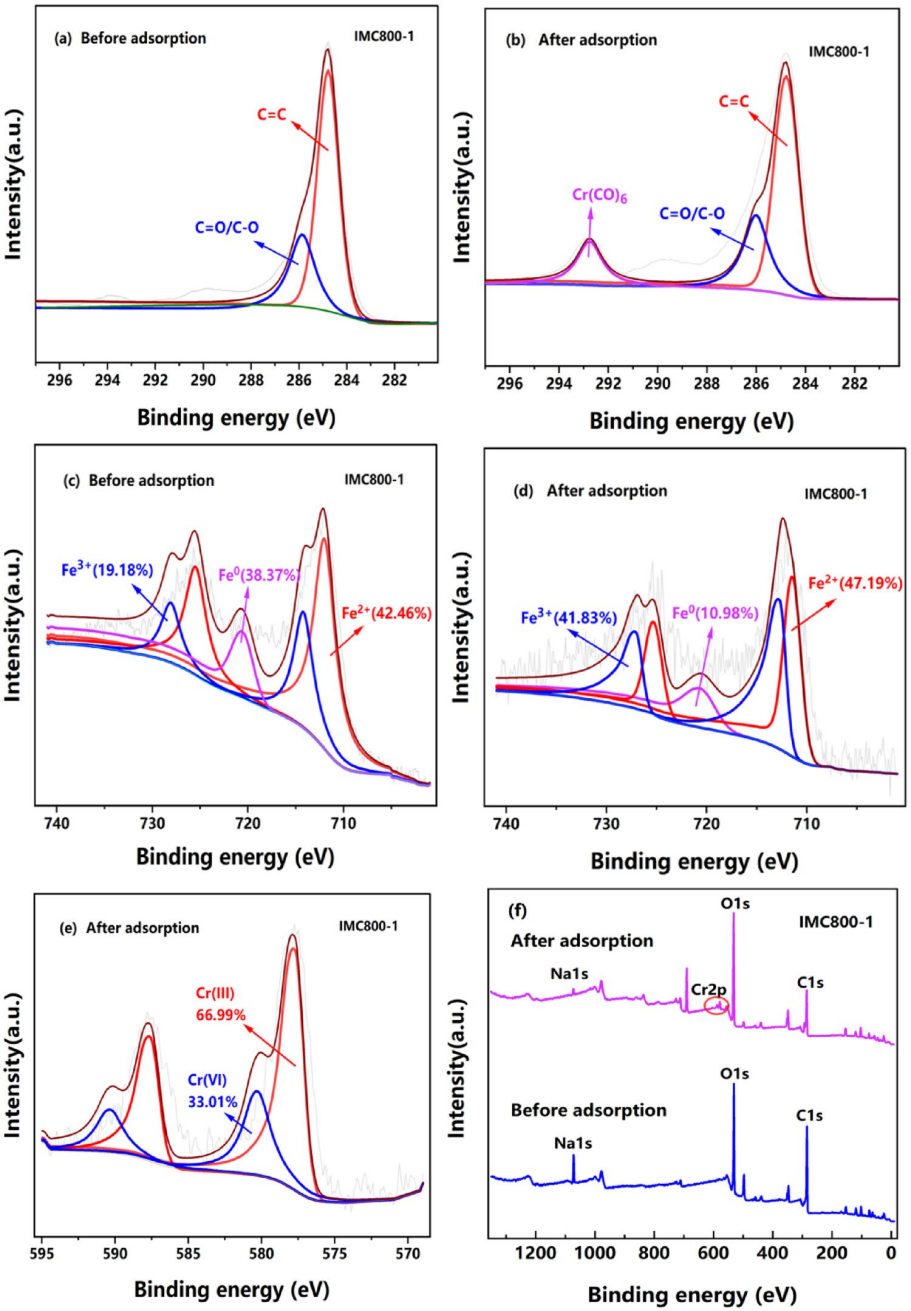
XPS spectra of IMC800-1 before and after Cr(VI) adsorption: (**a**), (**b**) C 1 s, (**c**), (**d**) Fe 2p, (**e**) Cr 2p, (**f**) Full spectra.

The chemical states of surface elements were comprehensively analyzed by XPS before and after adsorption. As shown in Fig. 6a,b, the C 1 s spectra with the binding energy of 284.52, 285.91, and 288.13 eV, which was associated with the presence of C=C, C=O/C–O, and Cr(CO)_6_, respectively^47^. These results elucidated that Cr element was successfully adhered by IMC. The appearance of Cr 2p in the whole spectra also confirms that Cr was removed by adsorbent, as presented in Fig. 6e,f. Meanwhile, as presented in Fig. 6c,d, the content of Fe(III) increased from 19.18 to 41.83% for IMC800-1, indicating the reactive Fe(II) and Fe^0^ species were oxidized during the Cr(VI) removal process. Interestingly, the ratio of Fe(II) almost retained or even increased, but the ratio of Fe^0^ decreased. for example, the contents of Fe(II) and Fe^0^ were 42.46% and 47.19%,38.37% and 10.98% for before and after adsorption, respectively. This phenomenon demonstrated that with the consumption of Fe(II) and Fe^0^ for Cr(VI) reduction, the Fe(III) and Fe^0^ could be reacted and consequently generated more Fe(II) species for the reaction^48^. The binding spectra of Cr(VI), with the binding energy ratio of 66.99% and 33.01% with the Cr(III) and Cr(VI), respectively, indicating Cr(VI) could be reduced into Cr(III). All these reaction processes could be explained as following Eqs. (3–6):

3 $${\text{Fe}}^{0} + {\text{O}}_{2} + 4{\text{H}}^{ + } \to 2{\text{Fe}}^{2 + } + 2{\text{H}}_{2} {\text{O }}$$

4 $${\text{Fe}}^{0} + {\text{HCrO}}_{4}^{ - } + 7{\text{H}}^{ + } \to {\text{Cr}}^{3 + } + {\text{Fe}}^{3 + } + 4{\text{H}}_{2} {\text{O }}$$

5 $$3{\text{Fe}}^{0} + {\text{Cr}}_{2} {\text{O}}_{7}^{2 - } + 14{\text{H}}^{ + } \to 2{\text{Cr}}^{3 + } + 3{\text{Fe}}^{2 + } + 7{\text{H}}_{2} {\text{O}}$$

6 $$3{\text{Fe}}^{2 + } + {\text{HCrO}}_{4}^{ - } + 7{\text{H}}^{ + } \to {\text{Cr}}^{3 + } + 3{\text{Fe}}^{3 + } + 4{\text{H}}_{2} {\text{O}}$$

**Figure 7:**
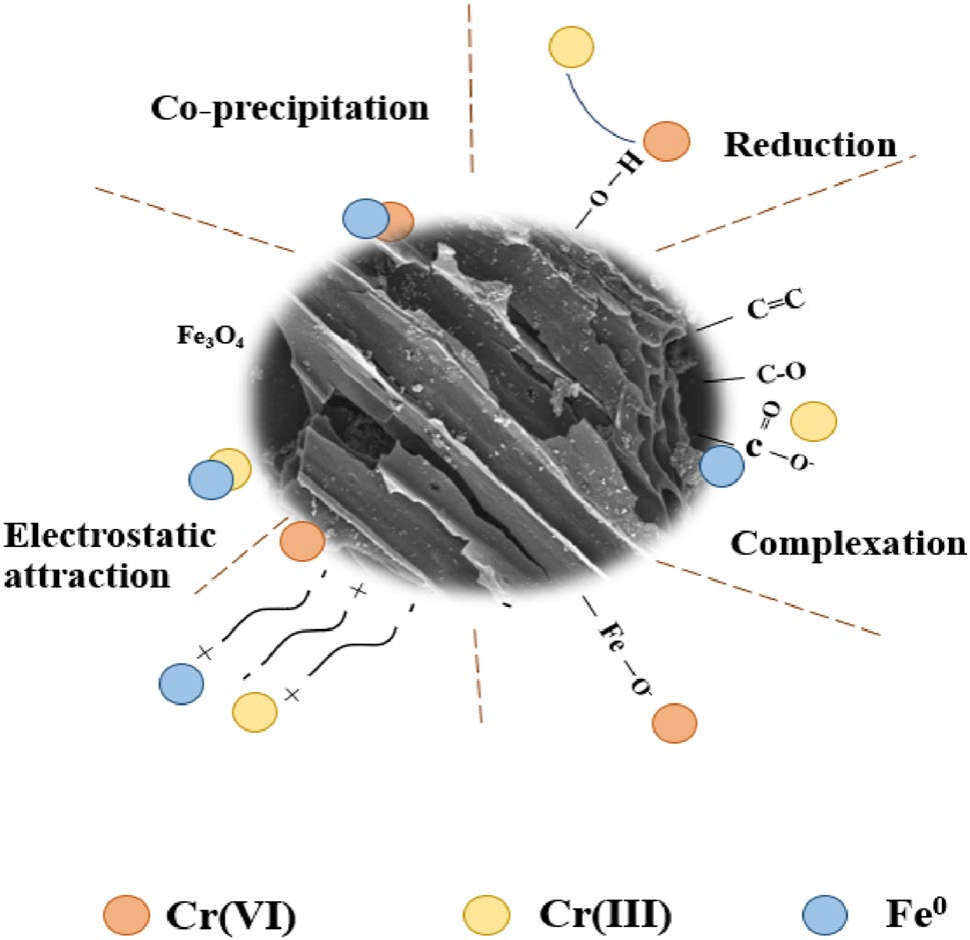
Adsorption mechanism of Cr(VI) by IMC.

As mentioned before, the mechanisms of Cr(VI) removal by IMC (Fig. 7) might be include chemical reduction, physical adherence, formation of complexations, co-precipitation.

We have made a comparison of Cr(VI) adsorption by different modified biochar, as indicated in the following Table 2.

**Table 2:** Comparison of parameters of different materials for Cr(VI) adsorption.

Adsorbent	Metal source	Preparation	Conditions	q_max_ (mg/g)	References
nZVI-RS	FeSO_4_·7H_2_O	600 °C, 2 h	pH 3.0–8.0	40.0	^50^
nZVI-HBC	FeSO_4_·7H_2_O	700 °C, 2 h	pH 3.0	96.0	^35^
S-nZVI/BC	FeCl·4H_2_O	400 °C, 3 h	pH2.5	126.0	^51^
SSB-nZVI	FeCl_2_·4H_2_O	NaBH_4_	pH 4.0	20.0	^52^
MMBC	Red mud	700 °C, 2 h	pH 3.0	99.8	^53^
IMC	Red mud, rice straw	800 °C, 1 h	pH 6.0	190.6	This work

## Conclusions

To sum up, the agro-industrial byproducts red mud and rice straw were applied as environmental benign and inexpensive raw materials for the integrated micro-electrolysis composites synthesis. The facile and time-saving hydrothermal one-pot method followed with pyrolysis processes. Further, the optimal preparation conditions were investigated and obtained, various techniques were employed to characterize the IMC, and IMC materials consisting of Fe0 species were successfully manufactured. Adsorption data of Cr(VI) by IMC were in good agreement with Langmuir isotherm model and pseudo-first-order kinetic model. Based on the thermodynamics results, the interactions fitted well with and, indicating the monolayer was endothermic spontaneous reaction. According to the batch experiments, IMC exhibited acceptable adsorption efficiency of 97.74%. The maximum removal capacity on Cr(VI) immobilization achieved 190.62 mg/g. All these results pave the way for a facile preparation and a new route to develop agro-industrial byproducts recycled functional materials, which is very important in the practical application for the heavy metals decontamination in wastewater.

## Materials and methods

### Chemicals and raw materials

The potassium dichromate (K_2_CrO_4_), hydrochloric acid (HCl, 36%), sodium hydroxide (NaOH) were purchased from Tianjin Zhiyuan Chemical Reagent Co. Ltd. All chemical reagents were analytical grade without further modification. Raw rice straw (RS) and red mud (RM) were obtained from Wenshan city, Yunnan province, China. The obtained RS was washed with DI water for several times and then dried at 60 °C in an oven for 24 h. After dried, RS was crushed and sieved through a 100 mesh sieve. The RM was smashed and dried in an oven (60 °C, 24 h) and then passed through the 100 mesh sieve before application. All chemicals were analytical grade without further purification.

### Preparation of IMC

Appropriate amounts of red mud and rice straw were added into the beaker with the mass ratios of 1:0.5, 1:1 and 1:2, respectively. 60 mL deionized water was added and put into the ultrasonic machine for 0.5 h. Then added 40 mL sodium hydroxide solution (0.1 M) and stirred for 2 h at room temperature (25 °C). The materials were put into an autoclave and the temperature was 120 °C for 10 h hydrothermal reaction, this process can closely combine the two substances; After cooling down, the suspension was poured out and dried in an oven with 80 °C for overnight. 15 g of the product was placed in a programmable tube furnace under the protection of N_2_. The heating rate was set as 5 °C/min, and the pyrolysis temperature was increased to 200 °C, 400 °C, 600 °C and 800 °C, respectively, and the holding time was 1 h to prepare zero-valent iron biochar complex. After cooling to room temperature, the samples were washed with deionized water several times until they reached neutral pH (7.0), then dried in a vacuum drying oven at 60 °C. The dried samples were put into sealed bags and marked for preservation.

### Characterizations

The crystalize structure of IMC was analyzed by X-ray diffraction instrument (XRD, Ultima IV, Nippon Science Company, Japan). Functional groups were examined by FTIR (Vertex 70, Bruker, Germany) ranging from 400 to 4000 cm^−1^ via a conventional KBr pellet method. The scanning electron microscope (SEM, Tescan mira4, Czech company) was applied to provide the IMC surface morphology. The chemical states of surface elements (Fe C and Cr) were confirmed by XPS (Thermo-Kalpha, America) analysis, and the C 1 s peaks at 284.8 eV was applied as background to calibrate the results. The surface charge of adsorbents was presented as zeta-potential and tested by the zeta sizer with pH ranging from 3 to 9.

### Batch experiments

The concentration of Cr(VI) was detected by UV–Vis coupled with 1,5-diphenyl carbazide method with the testing wavelength of 540 nm. Without special emphasis, in the subsequent adsorption experiment of Cr(VI), the concentration of Cr(VI) solution was 100 mg/L, the dosage of IMC was 20 mg. Each experiment data was sample three times as a parallel experiment, and until the corresponding errors were below 5%, to ensure the homoscedasticity and normality of the experimental data. In order to find a suitable dosage, 10–50 mg IMC were applied and initial Cr(VI) concentration was set at 100 mg/L with 12 h reaction time, the mixed solutions were shaken at a speed of 200 rpm under the temperature of 25 °C. The initial solution varying the pH from 2.0 to 7.0 was adjusted to desired value using 0.1 M NaOH or HCl solution. To analysis the physiochemical interactions between Cr(VI) and IMC, adsorption isotherms were conducted by changing the Cr(VI) initial concentration from 10 to 1000 mg/L, at dosage of adsorbent (40 mg), and the initial solution pH (6.0). The mixture was shaken at 298 K,308 K,318 K for 24 h to ensure the reaction reached equilibrium, respectively. The experimental data of adsorption isotherms were described by the Langmuir model and Freundlich model as listed in the following Eqs. (7) and (8):

7 $$q_{e} = \frac{{k_{L} q_{max} C_{e} }}{{1 + k_{L} C_{e} }}$$

8 $$q_{e} = K_{F} C_{e}^{\frac{1}{n}}$$

where q_e_ (mg/g) is the Cr(VI) amount adsorbed equilibrium adsorption capacity, q_max_ (mg/g) is the maximum adsorption capacity, k_L_ and k_F_ are the rate constants of pseudo-first-order model and pseudo-second-order model, respectively.

Furthermore, 20 mg adsorbents was dispersed into 10 mL solution contained Cr(VI) with the concentration of 200 mg/L for the kinetics study. Experimental data of kinetics study were fitted by pseudo-first-order model and pseudo-second-order model as listed in the following Eqs. (9) and (10):

9 $$q_{t} = q_{e} \left( {1 - e^{{tk_{1} }} } \right)$$

10 $$q_{t} = \frac{{k_{2} q_{e}^{2} t}}{{1 + k_{2} q_{e} t}}$$

where q_t_ (mg/g) is the Cr(VI) amount adsorbed on the surface of IMC at time t (min), q_e_ (mg/g) is the Cr(VI) amount adsorbed equilibrium adsorption capacity, k_1_ and k_2_ are the rate constants of pseudo-first-order model and pseudo-second-order model, respectively.

The thermodynamic parameters such as standard free energy (Δ*G*^0^), enthalpy (Δ*H*^0^) and entropy (Δ*S*^0^) and from 303 to 333 K in the adsorption processes were calculated according to Eqs. (11–13)^49^:

11 $$\Delta G^\circ = - RT\ln K$$

12 $$\ln K = \frac{{Q_{e} }}{{C_{e} }}$$

13 $$\ln K = \frac{{\Delta S^{^\circ } }}{R} - \frac{{\Delta H^{^\circ } }}{RT}$$

where R and T are the gas constant (8.314 J/(mol K^−1^)) and absolute temperature (K), respectively.

## Data Availability

All data generated or analyzed in this study are included in this published article.

## References

[1] Zhou X (2021). Tunable S doping from Co3O4 to Co9S8 for peroxymonosulfate activation: Distinguished Radical/Nonradical species and generation pathways. Appl. Catal. B..

[2] Ajiboye TO, Oyewo OA, Onwudiwe DC (2021). Simultaneous removal of organics and heavy metals from industrial wastewater: A review. Chemosphere.

[3] Hong Y (2019). Efficient degradation of atrazine by CoMgAl layered double oxides catalyzed peroxymonosulfate: Optimization, degradation pathways and mechanism. Chem. Eng. J..

[4] Kong L (2022). Simultaneous reduction and sequestration of hexavalent chromium by magnetic β-Cyclodextrin stabilized Fe3S4. J. Hazard. Mater..

[5] Plummer S (2018). Optimization of strong-base anion exchange O&M costs for hexavalent chromium treatment. Water Res..

[6] Yu R (2021). A high-efficiency Klebsiella variicola H12-CMC-FeS@biochar for chromium removal from aqueous solution. Sci Rep..

[7] Marinho BA, Cristóvão RO, Loureiro JM, Boaventura RAR, Vilar VJP (2016). Solar photocatalytic reduction of Cr(VI) over Fe(III) in the presence of organic sacrificial agents. Appl. Catal. B..

[8] Irfan M (2021). Heavy metals immobilization and improvement in maize (*Zea mays* L.) growth amended with biochar and compost. Sci. Rep..

[9] Krishna Kumar AS (2022). Heavy metal and organic dye removal via a hybrid porous hexagonal boron nitride-based magnetic aerogel. npj Clean Water..

[10] Yidong Z (2016). Environmental remediation and application of nanoscale zero-valent iron and its composites for the removal of heavy metal ions: A review. Environ. Sci. Technol..

[11] Wan Z (2019). Concurrent adsorption and micro-electrolysis of Cr(VI) by nanoscale zerovalent iron/biochar/Ca-alginate composite. Environ. Pollut..

[12] Wang P (2022). Use of sponge iron as an indirect electron donor to provide ferrous iron for nitrate-dependent ferrous oxidation processes: Denitrification performance and mechanism. Bioresour. Technol..

[13] de Souza Souza C (2021). Induced changes of pyrolysis temperature on the physicochemical traits of sewage sludge and on the potential ecological risks. Sci. Rep..

[14] Godlewska P, Ok YS, Oleszczuk P (2020). The dark side of black gold: Ecotoxicological aspects of biochar and biochar-amended soils. J. Hazard. Mater..

[15] Khurshid H, Mustafa MRU, Isa MH (2022). Adsorption of chromium, copper, lead and mercury ions from aqueous solution using bio and nano adsorbents: A review of recent trends in the application of AC, BC, nZVI and MXene. Environ. Res..

[16] Sun Y (2019). Multifunctional iron-biochar composites for the removal of potentially toxic elements, inherent cations, and hetero-chloride from hydraulic fracturing wastewater. Environ. Int..

[17] Wei D (2020). Simultaneous adsorption and oxidation of antimonite onto nano zero-valent iron sludge-based biochar: Indispensable role of reactive oxygen species and redox-active moieties. J. Hazard. Mater..

[18] Dongmei M (2021). Zero-valent iron and biochar composite with high specific surface area via K2FeO4 fabrication enhances sulfadiazine removal by persulfate activation. Chem. Eng. J..

[19] He Y (2022). Sewage-sludge derived activated carbon impregnated with polysulfide-sulfidated nZVI: A promising material for Cr(VI) reductive stabilization. Colloids Surf. Physicochem. Eng. Aspects.

[20] Wu W (2021). Red mud for the efficient adsorption of U(VI) from aqueous solution: Influence of calcination on performance and mechanism. J. Hazard. Mater..

[21] Yoon K (2019). Synthesis of functionalised biochar using red mud, lignin, and carbon dioxide as raw materials. Chem. Eng. J..

[22] Xiaohang Y (2022). One-pot preparations of cyclodextrin polymer-entrapped nano zero-valent iron for the removal of p-nitrophenol in water. Chem. Eng. J..

[23] Lui J, Chen W-H, Tsang DCW, You S (2020). A critical review on the principles, applications, and challenges of waste-to-hydrogen technologies. Renew. Sust. Energ. Rev..

[24] Liang J (2020). Rapid granulation using calcium sulfate and polymers for refractory wastewater treatment in up-flow anaerobic sludge blanket reactor. Bioresour. Technol..

[25] Wang M, Liu X (2021). Applications of red mud as an environmental remediation material: A review. J. Hazard. Mater..

[26] Olszewska JP, Heal KV, Winfield IJ, Eades LJ, Spears BM (2017). Assessing the role of bed sediments in the persistence of red mud pollution in a shallow lake (Kinghorn Loch, UK). Water Res..

[27] Zhou Y (2019). Applications of nanoscale zero-valent iron and its composites to the removal of antibiotics: A review. J. Membr. Sci..

[28] Zhou H (2022). Sodium citrate and biochar synergistic improvement of nanoscale zero-valent iron composite for the removal of chromium () in aqueous solutions. J. Environ. Sci..

[29] Kwon G (2022). Beneficial use of Fe-impregnated bentonite as a catalyst for pyrolysis of grass cut into syngas, bio-oil and biochar. Chem. Eng. J..

[30] Wen J, Fu W, Ding S, Zhang Y, Wang W (2022). Pyrogallic acid modified nanoscale zero-valent iron efficiently removed Cr(VI) by improving adsorption and electron selectivity. Chem. Eng. J..

[31] Chen H (2022). Insights into simultaneous adsorption and oxidation of antimonite [Sb(III)] by crawfish shell-derived biochar: Spectroscopic investigation and theoretical calculations. Biochar..

[32] Cui L (2021). Changes in surface characteristics and adsorption properties of 2,4,6-trichlorophenol following Fenton-like aging of biochar. Sci Rep..

[33] Zhang X (2022). Hydrochar magnetic adsorbent derived from Chinese medicine industry waste via one-step hydrothermal route: Mechanism analyses of magnetism and adsorption. Fuel.

[34] Xu Z, Xu X, Tsang DCW, Cao X (2018). Contrasting impacts of pre- and post-application aging of biochar on the immobilization of Cd in contaminated soils. Environ. Pollut..

[35] Zhu Y (2018). Removal of hexavalent chromium from aqueous solution by different surface-modified biochars: Acid washing, nanoscale zero-valent iron and ferric iron loading. Bioresour. Technol..

[36] Li Y (2022). High performance removal of sulfamethoxazole using large specific area of biochar derived from corncob xylose residue. Biochar.

[37] Pang Y (2022). Cadmium adsorption performance and mechanism from aqueous solution using red mud modified with amorphous MnO2. Sci Rep..

[38] Harikrishnan R, Mani G, Mani M, Kaviyarasu K, Baskaran I (2022). One step microwave assisted synthesis of praseodymium orthoferrite nanoparticles: Rietveld refinement phase matching, optical, and magnetic property analysis. Physica B.

[39] Zhao R (2022). Insights into Cr(VI) removal mechanism in water by facile one-step pyrolysis prepared coal gangue-biochar composite. Chemosphere.

[40] Dandan D, Oh KC, Jae WL (2022). Influence of the continuous addition of zero valent iron (ZVI) and nano-scaled zero valent iron (nZVI) on the anaerobic biomethanation of carbon dioxide. Chem. Eng. J..

[41] Shakeel A, Xiaomei L, Jingchun T, Shicheng Z (2022). Biochar-supported nanosized zero-valent iron (nZVI/BC) composites for removal of nitro and chlorinated contaminants. Chem. Eng. J..

[42] Dong H (2017). Stabilization of nanoscale zero-valent iron (nZVI) with modified biochar for Cr(VI) removal from aqueous solution. J. Hazard. Mater..

[43] Movasaghi Z, Yan B, Niu C (2019). Adsorption of ciprofloxacin from water by pretreated oat hulls: Equilibrium, kinetic, and thermodynamic studies. Ind. Crops Prod..

[44] Chen X, Li X, Xu D, Yang W, Bai S (2020). Application of nanoscale zero-valent iron in hexavalent chromium-contaminated soil: A review. Nanotechnol. Rev..

[45] Chen L (2018). Mechanisms of shale gas adsorption: Evidence from thermodynamics and kinetics study of methane adsorption on shale. Chem. Eng. J..

[46] Chen C (2021). Synthesis of zero-valent iron/biochar by carbothermal reduction from wood waste and iron mud for removing rhodamine B. Environ. Sci. Pollut. Res..

[47] Fan H (2020). High-gravity continuous preparation of chitosan-stabilized nanoscale zero-valent iron towards Cr(VI) removal. Chem. Eng. J..

[48] Wang H (2020). Black liquor as biomass feedstock to prepare zero-valent iron embedded biochar with red mud for Cr(VI) removal: Mechanisms insights and engineering practicality. Bioresour. Technol..

[49] Yang A, Wang Z, Zhu Y (2021). Facile preparation and highly efficient sorption of magnetic composite graphene oxide/Fe3O4/GC for uranium removal. Sci. Rep..

[50] Qian L (2017). Nanoscale zero-valent iron supported by biochars produced at different temperatures: Synthesis mechanism and effect on Cr(VI) removal. Environ. Pollut..

[51] Gao J (2018). Scavenging of Cr(VI) from aqueous solutions by sulfide-modified nanoscale zero-valent iron supported by biochar. J. Taiwan Inst. Chem. Eng..

[52] Diao ZH (2018). Insights into the simultaneous removal of Cr(6+) and Pb(2+) by a novel sewage sludge-derived biochar immobilized nanoscale zero valent iron: Coexistence effect and mechanism. Sci. Total Environ..

[53] Cho DW (2019). Fabrication and environmental applications of multifunctional mixed metal-biochar composites (MMBC) from red mud and lignin wastes. J. Hazard. Mater..

